# Updates in Hemoglobinopathies

**DOI:** 10.1155/2013/912913

**Published:** 2013-10-03

**Authors:** Youssef Al-Tonbary, Fernando Tricta, Amal El-Beshlawy, Mohamed Ahmed Badr, Ahmed Mansour

**Affiliations:** ^1^Department of Pediatric Hematology and Oncology, Mansoura University, Mansoura 35516, Egypt; ^2^Department of Pediatric Hematology and Oncology, ApoPharma, Toronto, ON, Canada; ^3^Department of Pediatric Hematology, Cairo University, Cairo 12311, Egypt; ^4^Department of Pediatric Hematology and Oncology, Zagazig University, Zagazig 05533, Egypt

I am honored to invite you to enjoy reading this special issue of The Scientific World Journal.

One review article is “*Ineffective erythropoiesis in *β*-thalassemia*.” The authors concluded that this ineffective erythropoiesis could be the conjunction of several mechanisms of which the final consequence is the arrest of maturation and increased apoptosis of erythroblasts during their terminal differentiation stage.

Another review article discusses the biologic complexity in sickle cell disease. This complexity is likely to be one of the major barriers to the development of successful new treatments which, to date, has largely concentrated on individual mechanistic pathways. Future development of therapeutics needs to continue.

Another review article titled “*Phytomedicines and nutraceuticals: alternative therapeutics for sickle cell anemia*” highlights the feasibility of botanicals, mainly antisickling phytomedicines and nutraceuticals, as attractive potential candidates for sickle cell anemia therapy and strongly collaborates the ethnomedical usage of the plants.

A peer-reviewed original article was carefully selected from many articles submitted to the journal. It studied the impact of migrations on the health services for hemoglobin disorders in Europe. Its results show that countries with traditional strong prevention and treatment programs are well prepared to face these challenges, while others are urgently needed to address these problems in a systematic way.

More articles will be published in this special issue. On behalf of the guest editors of this special issue of The Scientific World Journal, I look forward to receiving your comments and any suggestions you may have. 

*Youssef Al-Tonbary* *Youssef Al-Tonbary* *Fernando Tricta* *Fernando Tricta* *Amal El-Beshlawy* *Amal El-Beshlawy* *Mohamed Ahmed Badr* *Mohamed Ahmed Badr* *Ahmed Mansour* *Ahmed Mansour*

